# Fluorescence microscopy visualization of halomucin, a secreted 927 kDa protein surrounding *Haloquadratum walsbyi* cells

**DOI:** 10.3389/fmicb.2015.00249

**Published:** 2015-03-30

**Authors:** Ralf Zenke, Susanne von Gronau, Henk Bolhuis, Manuela Gruska, Friedhelm Pfeiffer, Dieter Oesterhelt

**Affiliations:** ^1^Imaging Facility, Max-Planck-Institute of BiochemistryMartinsried, Germany; ^2^Department of Membrane Biochemistry, Max-Planck-Institute of BiochemistryMartinsried, Germany; ^3^Yerseke Marine Microbiology, Royal Netherlands Institute for Sea ResearchYerseke, Netherlands; ^4^Department of Molecular Structural Biology, Max-Planck-Institute of BiochemistryMartinsried, Germany

**Keywords:** halomucin, halophilic archaea, polyhydroxybutyrate, cell shape, *Haloquadratum*, immunofluorescence, protein secretion

## Abstract

At the time of its first publication, halomucin from *Haloquadratum walsbyi* strain HBSQ001 was the largest archaeal protein known (9159 aa). It has a predicted signal sequence, making it likely to be an extracellular or secreted protein. Best BLAST matches were found to be mammalian mucins that protect tissues to dehydration and chemical stress. It was hypothesized that halomucin participates in protection against desiccation by retaining water in a hull around the halophilic organisms that live at the limits of water activity. We visualized *Haloquadratum* cells by staining their intracellular polyhydroxybutyrate granules using Nile Blue. Halomucin was stained by immunofluorescence with antibodies generated against synthetic peptides derived from the halomucin amino acid sequence. Polyhydroxybutyrate stained cells were reconstructed in 3D which highlights not only the highly regular square shape but also the extreme flatness of *Haloquadratum*. Double-staining proves halomucin to be extracellular but to be only loosely associated to cells in agreement with its hypothesized function.

## Introduction

Extreme hypersaline ecosystems with NaCl concentrations approaching saturation harbor a rich microbial community which is dominated by halophilic archaea of the family Halobacteriaceae (Oren, 2002, 2012). The dominant organism found at the highest salinities is a square gas-vesicle containing archaeon that was first detected by Walsby (1980). In many laboratories attempts have been undertaken to grow these spectacular organisms in pure culture but even in 2002, more than two decades after the initial description, Oren writes that “At present, microbiologists still dream of growing and characterizing the square, flat, halophilic Archaeon, first described in 1980…” (Oren, 2002).

At that time, a pleomorphic halophilic archaeon (strain 801030/1) had been cultivated which included square cells but lacked the characteristic gas vesicles described by Walsby. This isolate is now being known as *Haloarcula quadrata* (Oren et al., 1999; Oren, 2002). It is motile and contains flagella that form a right-handed helix as previously described in an environmental sample (Alam et al., 1984).

After attempts to grow Walsby's square archaeon had failed for more than 2 decades, two groups independently reported the cultivation of Walsby's square archaeon in 2004 (Bolhuis et al., 2004; Burns et al., 2004). The obtained isolates are so similar in their genetic makeup that they are now recognized as strains from the same species, *Haloquadratum walsbyi* (Burns et al., 2007), a name which highlights the hypersalinity tolerated by the organism, its peculiar shape, and honors the scientist who first described the organism (Walsby, 1980). *Hqr. walsbyi* can grow in media which are not only saturated in salt (3.3 M NaCl) but in addition contain more than 2 M MgCl_2_ (Bolhuis et al., 2004). This has been referred to as “life at the limits of water activity” (Bolhuis et al., 2006). Despite these extreme conditions, *Haloquadratum* reaches very high cell densities (Walsby, 1980; Oren, 2002) but its growth is exceedingly slow (Bolhuis et al., 2004; Burns et al., 2004).

The Spanish isolate is strain HBSQ001, the Australian isolate is strain C23 and is the type strain of the organism (Burns et al., 2007). The two strains show many similarities but some strain-specific features were detected, including a different number of the cell surface layers (Burns et al., 2007). The genome sequence proved highly similar (98.6% sequence identity, including intergenic regions, and a complete lack of genome rearrangements) (Dyall-Smith et al., 2011) even though the sampling sites (Spain, Australia) represent near-maximal distance on Earth. This confirmed the close similarity already inferred from ecological data and was interpreted as “limited diversity in a global pond” (Dyall-Smith et al., 2011).

Two fascinating aspects of *Hqr. walsbyi* are its square shape and its extreme flatness (about 0.1–0.5 μm) (Walsby, 1980; Stoeckenius, 1981; Kessel and Cohen, 1982; Bolhuis et al., 2004; Burns et al., 2007). If an organism attempts to increase the surface to volume ratio, it has two options: (i) reduction in size (which is only possible up to certain limits) or (ii) to become flat, thus avoiding a limitation of cell size and leading to square cells upon cell-division (Bolhuis, 2005). *Haloquadratum* cells range in size from 1.5 to 11 μm (Walsby, 1980) but extremely large cells of more than 40 × 40 μm have been observed with no indication of cell division structures (Bolhuis et al., 2004). Tomography images show that Haloquadratum has two types of intracellular structures: gas vesicles (e.g., gvpA, HQ_1782A) and polyhydroxybutyrate (PHB) granules. The latter can be visualized with the fluorescent dye Nile Blue A (Ostle and Holt, 1982). The biosynthetic enzymes have been identified in the genome (phaBCE: HQ_2309A-HQ_2311A) (Bolhuis et al., 2006; Dyall-Smith et al., 2011). It were actually the gas vesicles that alerted Walsby of the square cells which otherwise would have been easily overlooked (Walsby, 1980).

One of the highlights found in the genome of the Spanish isolate of *Hqr. walsbyi* was halomucin, the largest archaeal protein known at that time (9159 residues, 927 kDa). Among the best matches in databases were the sequences of vertebrate mucin and it was proposed that halomucin forms a water enriched capsule around the cells. This prediction is based on the presence of a typical Sec-type signal sequence at the N-terminus of the protein. Interestingly, the ortholog from strain C23 (Hqrw_1107) is significantly shorter (7836 aa) and lacks the two copies of the CTLD (C-type lectin domain) (InterPro:IPR001304) that are found in the protein from strain HBSQ001 (Dyall-Smith et al., 2011).

Mucins form important structures in prokaryotes and eukaryotes including mammals where they are involved in protection of cells or cell tissues to desiccation or chemical stress (Tabak et al., 1982). Mucins also have been implicated in the protection of mammalian tissues against viral attack and pathogenic bacteria and may be applied in purified form as a broad-range antiviral supplement to personal hygiene products, baby formula or lubricants to support the human immune system (Lieleg et al., 2012).

Secretion of such a huge protein through the Sec core seems challenging. We wanted to confirm that halomucin is a secreted protein and wanted to investigate the type of association between halomucin and its cells. We addressed this by double-staining where cells were visualized by Nile Blue staining of their cytoplasmic polyhydroxybutyrate granules and halomucin was visualized by immunostaining. We found it to be an extracellular protein which is only loosely attached to cells.

## Materials and methods

For staining of polyhydroxybutyrate granules, a Nile Blue stock solution in ethanol was prepared (1 mg/ml). *Hqr. walsbyi* strain HBSQ001 was grown in HAS medium (with CaCl_2_ reduced to 0.3 mM) to stationary phase as described (Bolhuis et al., 2004). 100 μl of cells were mixed with 100 μl HAS medium (Bolhuis et al., 2004) and 6 μl of the Nile Blue stock solution and incubated for at least 1 h. Cells were visualized in a Leica SP2 confocal microscope, excitation (Nile Blue): 633 nm, detection: 645–750 nm.

Polyclonal antibodies against halomucin were generated in chicken to allow subsequent usage under high-salt conditions. Antibodies were generated using synthetic peptides as antigen (Peptide synthesis at MPI of Biochemistry, Core Facility). The synthetic peptides with the unique amino acid sequences, NELSVDTSAPQIDDLSA and RGAAPAWLGVVSGPAATA, corresponded to position 1458–1474 and 8774–8791 of HQ_1081A, respectively. Polyclonal antibodies were generated by Davids Biotechnologie, Regensburg, Germany. Prior to use, polyclonal antibodies were diluted (1:5, 1:25, 1:100) with HAS medium.

For fluorescent microscopy, a FITC-conjugated rabbit-anti-chicken-IgY (Promega #G2691, Promega, Madison, WI) secondary antibody was used. This antibody was diluted (1:6) with HAS medium.100 μl *Hqr. walsbyi* cells were mixed with 100 μl diluted polyclonal antibody and incubated for 1 h at room temperature. Then, 20 μl of the secondary antibody dilution were added. For double staining, 6 μl of the Nile Blue stock solution were added. Cells were visualized by Leica Confocal Software.

## Results and discussion

**Figure 1:**
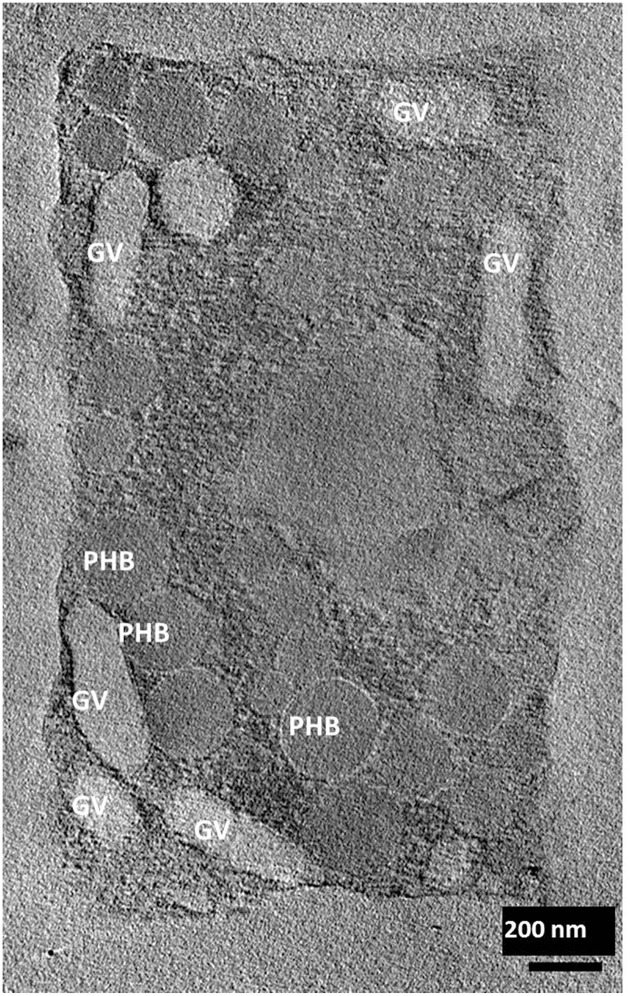
**Electron tomographic image of a single square cell of *Hqr. walsbyi* (reproduced from Bolhuis et al., 2006)**. Gas vesicles (GV) and polyhydroxybutyrate granules (PHB) are indicated (image by M. Gruska while working with H. Engelhardt).

**Figure 2:**
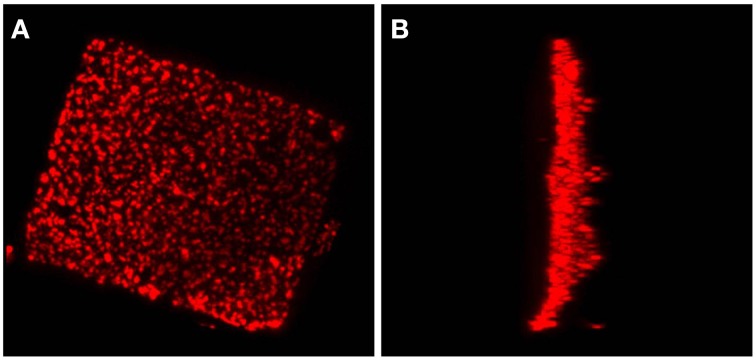
**A *Haloquadratum* cell with polyhydroxybutyrate granules stained by Nile Blue**. This figure shows a top view **(A)** and a side view **(B)** from a 3D reconstruction based on a z-stack series of fluorescence microscopic images. An animated version of this 3D reconstruction is found in the Supplementary Material (Video S1).

Electron tomography imaging of a single square cell of *Hqr. walsbyi* strain HBSQ001 revealed the presence of gas vesicles (GV) and PHB granules (Figure 1, reproduced from Bolhuis et al., 2006). The contours of the cells were visualized by staining the ubiquitously present polyhydroxybutyrate granules with Nile Blue and imaged by fluorescence microscopy (Figure 2). This image shows the head-on and side-on views of a single cell, confirming the square shape and the extreme flatness of *Hqr. walsbyi* (Walsby, 1980; Stoeckenius, 1981; Bolhuis, 2005; Burns et al., 2007). Figures 2A,B are stills from a short video clips (Video S1) where the 3D reconstructed cells are animated to represent one complete 360° turn of the cell. Several such video clips are available in the Supplementary Material (Videos S1–S4). Once more, these images confirm the extremely unusual cell shape of *Hqr. walsbyi*. Cells from this organism are distinctively square, while those from other species like *Haloarcula quadrata*, which do form quadratic cells, appear overall more irregular (Oren et al., 1999). *Hqr. walsbyi* cells are extremely flat (ca 0.2 μm) whereas the length in the other two dimensions are at least 5 μm. The extreme flatness of the cells may imply the complete absence of cell turgor but the forces that cause cell edges to be as straight as they are observed are currently enigmatic since no genes could be identified in the genome that might express structural proteins involved in maintaining the cell structure. It has been argued that the corners are rather a secondary consequence of the flat morphology (Bolhuis, 2005). In prokaryotic cells many essential processes take place at their surface. These include amongst others nutrient and oxygen uptake, light driven generation of a transmembrane proton gradient and extrusion of end products. The large flat cells of *Hqr. walsbyi* guarantee the perfect surface to volume ratio and allow cells to become very large without suffering the drawbacks of limited diffusion speed of essential nutrients as in spherical cells.

**Figure 3:**
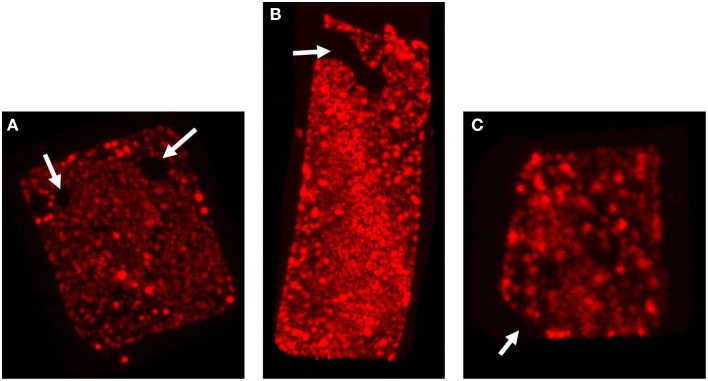
**Irregular cell shapes in *Hqr. walsbyi***. The three images show the top view of different Nile Blue stained cells. The white arrows illustrate the different irregularities as discussed in the text: **(A)** “holes” in the cell, **(B)** a rectangular cell, also containing a more extended stain-free region, and **(C)** a cell where one corner has a more “edged” shape. Each of the images corresponds to one of the animations provided in the Supplementary Material (Videos S2–S4).

Although the majority of the cells are distinctively square, sometimes deviations in cell shape can be observed as shown in Figure 3. The cell in Figure 3A contains round, circular, stain-free regions that appear as holes. Similar “holes” or electron dense regions have been observed before (Stoeckenius, 1981). Currently there is no satisfactory explanation for these structures but these might be caused by either damage of the fragile cells, presence of a high density material like DNA or regions where the opposing cell membranes are in contact, thereby not leaving any space for gas vesicles or PHB granules. In Figure 3B the cell appears rectangular rather than quadratic and is most likely an example of an elongating cell before cell division. This cell contains a more extended stain-free region but still seems intact as it retains the PHB granules in the cytoplasm. In Figure 3C only three of the four edges form a perfect straight corner whereas the fourth corner has a more “edged” shape. Also these edged parts have been observed previously but may simply express the fragility of these square cells, especially when treated with stains and undergoing washing steps before microscopy.

**Figure 4:**
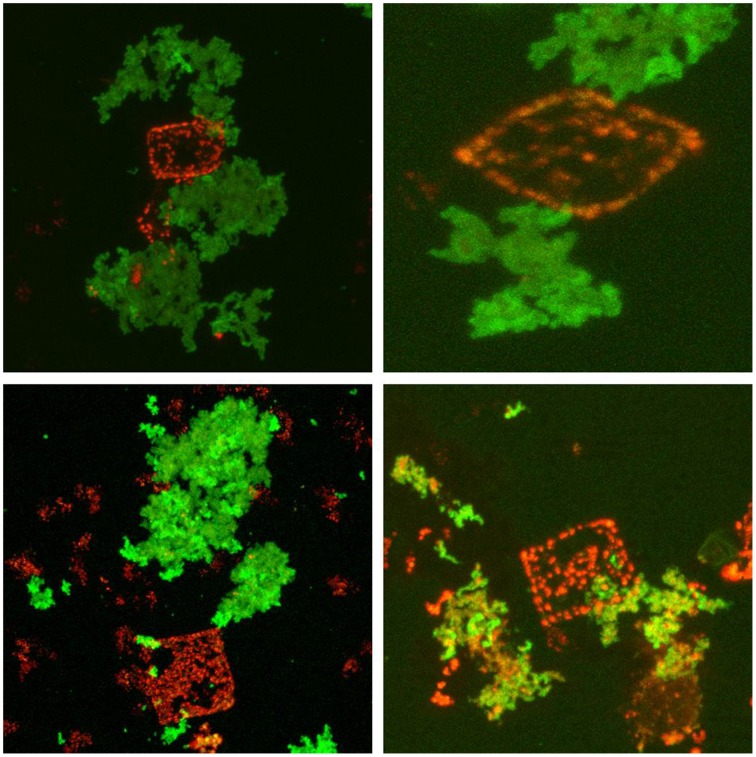
**Immunofluorescence stained halomucin loosely associated with the Nile Blue stained *Hqr. walsbyi* cells**. Immunofluorescence staining appears green while Nile Blue staining of polyhydroxybutyrate granules appears red and marks *Haloquadratum* cells. Halomucin is clearly an extracellular protein which is only found loosely attached to cells.

It has been predicted that halomucin is a secreted protein based on the detection of a Sec-type signal sequence at the N-terminus of the protein. However, a protein that huge may be a challenge for any secretion system and true evidence for the extracellular location of halomucin was lacking. That the electron tomographical image in Figure 1 does no reveal any features that can be interpreted as extracellular mucin is most likely caused by the fact that the preparations required for this type of images is often destructive to the extracellular features. Of all the images of the square archaeon that are published since its discovery in 1980, only one electron microscopy image reveals an extracellular matrix (Kessel and Cohen, 1982) and only after the identification of halomucin in the genome of *Hqr. walsbyi*, it was speculated that this matrix might be the illusive halomucin (Bolhuis et al., 2004). Using the polyclonal antibodies generated against two synthetic peptides derived from the halomucin sequence we could for the first time successfully identify halomucin (Figure 4). The applied double-staining technique clearly identified the halomucin outside the cells proving that indeed this huge protein is secreted over the cell membrane. As proteins are normally transported in their unfolded state through the Sec pore of the membrane, this apparently is also true for the extremely long halomucin protein. Considering an average translocation rate of 270 amino acid residues per minute and an expense of one ATP per 50 amino acids as determined for the SecEYGA translocation system of *Escherichia coli* (Tomkiewicz et al., 2006), the translocation of one 9159 amino acids long halomucin protein would take about 34 min and require the hydrolysis of about 183 ATP molecules. This would be quite a burden on the protein translocation system but possibly only a few halomucin proteins are sufficient to exert their extracellular function thus preventing blockage of the secretory system. Halomucin was proposed to function as water enriched capsule around the cells although alternative functions such as involvement in generating a defensive barrier against cations or halophages might also be possible. Halomucin as a phage resistance barrier makes sense since phages are ubiquitous in salterns and the genome of *Hqr. walsbyi* shows sufficient evidence of phage related genes and DNA insertions in the past.

There are similarities in amino acid sequence and domain organization to mammalian mucins (Bolhuis et al., 2006) which are known to protect various tissues against desiccation or harsh chemical conditions. Halomucin may be important for *Hqr. walsbyi* to grow at the limits of water activity in a way that is reminiscent of the mucous lungfish cocoon, which enables it to survive for months outside of water (Chew et al., 2004). However, the sequence databases have grown considerably since the initial publication of the halomucin sequence. When comparing halomucin to these updated databases, a number of proteins of prokaryotic origin give a higher blast score than the initially identified mammalian mucins (Dyall-Smith et al., 2011).

The halomucin of the two isolated *Haloquadratum* strains show distinct differences, with the protein from strain C23 being significantly shorter (7836 aa) than the ortholog from strain HBSQ001 (9159 aa). This size reduction has been attributed to deletion of two internal gene segments of 2.4 kb (codons 283–1075) and 1.3 kb (codons 5078–5583) (Dyall-Smith et al., 2011). The protein regions selected for antibody production are present in both strains but only one (pos 877–8791) is identical in the two strains while the other (pos 1458–1474) is in a highly variable region (8 of 17 residues differ). A distinct difference is the lack of a pair of CTLD (C-type lectin domain) (InterPro:IPR001304) domains in strain C23, caused by the 2.4 kb deletion. It has been speculated that halomucin is modified by sialic acid (Bolhuis et al., 2006), a post-translational modification which is largely restricted to higher eukaryotic organisms. The corresponding pair of sialic acid biosynthesis proteins (neuAB, HQ_3518A/HQ_3519A) is, however, restricted to strain HBSQ001. The strain-specific occurrence of sialic acid biosynthesis genes and of the halomucin CTLD domains may indicate a functional correlation.

Upon double-staining, halomucin becomes visible in the form of large clusters which seem neither closely associated with the cells nor completely independent. At the point of closest association, halomucin may be connected/bound to the cell surface. It cannot be excluded that detachment of a halomucin cluster from the cell tears part of the cell surface away, leading to the observed “holes.” However, this would imply a distinct capacity of the cell for resealing of the cytoplasmic membrane as PHB granules are retained in the cytoplasm. It is more likely that in the natural environment, which is devoid of strong external disturbances, this loose association is sufficient to exert its protective functioning.

It should be noted that we applied the antibodies to intact cells without fixation or membrane permeabilization. Thus, an intracellular pool of halomucin, if present, would have escaped detection.

In conclusion, we have experimentally confirmed halomucin to be a secreted protein which is found outside of but only loosely connected to the cell. In addition, we provide a set of images and video films that highlight the spectacular shape of this organism which dominates the most salty ecological niches known to support biological growth on earth.

## Author contributions

DO designed the work. HB isolated and cultivated this strain of the organism and analyzed data. SG performed cell staining. RZ carried out microscopy, image analysis, and 3D reconstruction. MG generated the tomographic image of *Haloquadratum*. FP analyzed data. FP, HB, and DO wrote the manuscript.

### Conflict of interest statement

The authors declare that the research was conducted in the absence of any commercial or financial relationships that could be construed as a potential conflict of interest.

## References

[B1] Alam M. Claviez M. Oesterhelt D. Kessel M. (1984). Flagella and motility behaviour of square bacteria. EMBO J. 3, 2899–2903. 6526006 10.1002/j.1460-2075.1984.tb02229.x PMC557786

[B2] Bolhuis H. (2005). Walsby's square archaeon, in Adaptation to Life at High Salt Concentrations in Archaea, Bacteria, and Eukarya, eds Gunde-Cimerman N. Oren A. Plemenitas A. (Dordrecht: Springer), 185–199.

[B3] Bolhuis H. Palm P. Wende A. Falb M. Rampp M. Rodriguez-Valera F.. (2006). The genome of the square archaeon *Haloquadratum walsbyi*: life at the limits of water activity. BMC Genomics 7:169. 10.1186/1471-2164-7-169 16820047 PMC1544339

[B4] Bolhuis H. te Poele E. M. Rodriguez-Valera F. (2004). Isolation and cultivation of Walsby's square archaeon. Environ. Microbiol. 6, 1287–1291. 10.1111/j.1462-2920.2004.00692.x 15560825

[B5] Burns D. G. Camakaris H. M. Janssen P. H. Dyall-Smith M. L. (2004). Cultivation of Walsby's square haloarchaeon. FEMS Microbiol. Lett. 238, 469–473. 10.1111/j.1574-6968.2004.tb09790.x 15358434

[B6] Burns D. G. Janssen P. H. Itoh T. Kamekura M. Li Z. Jensen G.. (2007). *Haloquadratum walsbyi* gen. nov., sp. nov., the square haloarchaeon of Walsby, isolated from saltern crystallizers in Australia and Spain. Int. J. Syst. Evol. Microbiol. 57, 387–392. 10.1099/ijs.0.64690-0 17267984

[B7] Chew S. F. Chan N. K. Loong A. M. Hiong K. C. Tam W. L. Ip Y. K. (2004). Nitrogen metabolism in the African lungfish (*Protopterus dolloi*) aestivating in a mucus cocoon on land. J. Exp. Biol. 207, 777–786. 10.1242/jeb.00813 14747410

[B8] Dyall-Smith M. L. Pfeiffer F. Klee K. Palm P. Gross K. Schuster S. C.. (2011). *Haloquadratum walsbyi*: limited diversity in a global pond. PLoS ONE 6:e20968. 10.1371/journal.pone.0020968 21701686 PMC3119063

[B9] Kessel M. Cohen Y. (1982). Ultrastructure of square bacteria from a brine pool in Southern Sinai. J. Bacteriol. 150, 851–860. 7068535 10.1128/jb.150.2.851-860.1982 PMC216438

[B10] Lieleg O. Lieleg C. Bloom J. Buck C. B. Ribbeck K. (2012). Mucin biopolymers as broad-spectrum antiviral agents. Biomacromolecules 13, 1724–1732. 10.1021/bm3001292 22475261 PMC3597216

[B11] Oren A. (2002). Molecular ecology of extremely halophilic Archaea and Bacteria. FEMS Microbiol. Ecol. 39, 1–7. 10.1111/j.1574-6941.2002.tb00900.x 19709178

[B12] Oren A. (2012). Taxonomy of the family Halobacteriaceae: a paradigm for changing concepts in prokaryote systematics. Int. J. Syst. Evol. Microbiol. 62, 263–271. 10.1099/ijs.0.038653-0 22155757

[B13] Oren A. Ventosa A. Gutierrez M. C. Kamekura M. (1999). *Haloarcula quadrata* sp. nov., a square, motile archaeon isolated from a brine pool in Sinai (Egypt). Int. J. Syst. Bacteriol. 49, 1149–1155. 10.1099/00207713-49-3-1149 10425773

[B14] Ostle A. G. Holt J. G. (1982). Nile Blue A as a fluorescent stain for poly-beta-hydroxybutyrate. Appl. Environ. Microbiol. 44, 238–241. 6181737 10.1128/aem.44.1.238-241.1982 PMC241995

[B15] Stoeckenius W. (1981). Walsby's square bacterium: fine structure of an orthogonal procaryote. J. Bacteriol. 148, 352–360. 7287626 10.1128/jb.148.1.352-360.1981 PMC216199

[B16] Tabak L. A. Levine M. J. Mandel I. D. Ellison S. A. (1982). Role of salivary mucins in the protection of the oral cavity. J. Oral Pathol. 11, 1–17. 10.1111/j.1600-0714.1982.tb00138.x 6801238

[B17] Tomkiewicz D. Nouwen N. van Leeuwen R. Tans S. Driessen A. J. M. (2006). SecA supports a constant rate of preprotein translocation. J. Biol. Chem. 281, 15709–15713. 10.1074/jbc.M600205200 16601117

[B18] Walsby A. E. (1980). A square bacterium. Nature 283, 69–71 10.1038/283069a0

